# Preoperative Imaging Signs of Cerebral Malperfusion in Acute Type A Aortic Dissection: Influence on Outcomes and Prognostic Implications—A 20-Year Experience

**DOI:** 10.3390/jcm12206659

**Published:** 2023-10-20

**Authors:** Mohammed Al-Tawil, Mohamed Salem, Christine Friedrich, Shirin Diraz, Alexandra Broll, Najma Rezahie, Jan Schoettler, Nora de Silva, Thomas Puehler, Jochen Cremer, Assad Haneya

**Affiliations:** Department of Cardiovascular Surgery, University Hospital of Schleswig-Holstein, 24118 Kiel, Germanychristine.friedrich@uksh.de (C.F.); shirin.diraz@uksh.de (S.D.);

**Keywords:** type A aortic dissection, cerebral malperfusion, stroke

## Abstract

Background: Acute type A aortic dissection (ATAAD) continues to be a subject of active research due to its high mortality rates and associated complications. Cerebral malperfusion in ATAAD can have a devastating impact on patients’ neurological function and overall quality of life. We aimed to explore the risk profile and prognosis in ATAAD patients presenting with preoperative imaging signs of cerebral malperfusion (PSCM). Methods: We obtained patient data from our Aortic Dissection Registry, which included 480 consecutive ATAAD cases who underwent surgical repair between 2001 and 2021. Primary endpoint outcomes included the in-hospital and 30-day mortality, postoperative new neurological deficit, mechanical ventilation hours, and intensive care unit (ICU) length of stay. Results: Of the total cohort, 82 patients (17.1%) had PSCM. Both groups had similar distributions in terms of age, sex, and body mass index. The patients in the PSCM group presented with a higher logistic EuroSCORE (47, IQR [31, 64] vs. 24, IQR [15, 39]; *p* < 0.001) and a higher portion of patients with a previous cardiac surgery (7.3% vs. 2.0%; *p* = 0.020). Intraoperatively, the bypass, cardioplegia, and aortic cross-clamp times were similar between both groups. However, the patients in the PSCM group received significantly more intraoperative packed red blood cells, fresh frozen plasma, and platelets transfusions (*p* < 0.05). Following the surgery, the patients who presented with PSCM had markedly longer ventilation hours (108.5 h, IQR [44, 277] vs. 43 h, IQR [16, 158], *p* < 0.001) and a significantly longer ICU length of stay (7 days, IQR [4, 13] vs. 5 days, IQR [2, 11]; *p* = 0.013). Additionally, the patients with PSCM had significantly higher rates of postoperative new neurological deficits (35.4% vs. 19.4%; *p* = 0.002). In the Cox regression analysis, PSCM was associated with significantly poorer long-term survival (hazard ratio (HR) 1.75, 95%CI [1.20–2.53], *p* = 0.003). Surprisingly, hypertension was shown as a protective factor against long-term mortality (HR: 0.59, 95%CI [0.43–0.82], *p* = 0.001). Conclusion: PSCM in ATAAD patients is linked to worse postoperative outcomes and poorer long-term survival, emphasizing the need for early recognition and tailored management.

## 1. Introduction

Acute type A aortic dissection (ATAAD) continues to be a subject of active research due to its high mortality rates and associated perioperative complications. Despite the ongoing advancements in the diagnosis and expedited management of ATAAD, the early mortality rates remain alarmingly high. In contemporary practice, the in-hospital mortality rate for ATAAD patients who undergo surgery ranges between 10 and 18% with considerable between-hospital variations [1,2,3,4,5].

Still, the preoperative ATAAD profile is a major moderator of outcomes in those patients. A critical aspect that necessitates careful attention is when the dissection involves one of the vital organs’ perfusing vessels, causing malperfusion. The presence of preoperative malperfusion affecting cerebral, myocardial, mesenteric, spinal, renal, or lower limb regions significantly increases the risk of mortality and perioperative adverse events [6,7,8,9,10].

Among the various forms of malperfusion, cerebral malperfusion (CM) holds particular significance. CM can be indicated by preoperative imaging signs (PSCM) or intraoperatively, and can have a devastating impact on a patient’s neurological function, cognitive abilities, and overall quality of life. A recent systematic review estimated that CM occurs in approximately 16% of patients presenting with ATAAD and is associated with an in-hospital mortality rate of around 20% [11]. However, advancements in the early surgical intervention and expedited management of patients with CM have contributed to a substantial decrease in mortality rates in recent years [4,11].

The main objective of this study was to analyze the outcomes and investigate the impact of CM indicated by PSCM on the patient prognosis. Furthermore, we aimed to examine the differences in the various preoperative, intraoperative, and postoperative factors associated with prognosis.

## 2. Materials and Methods

### 2.1. Study Design and Patient Population

We obtained patient data from our Aortic Dissection Registry, which included 480 consecutive ATAAD cases who underwent surgical repair under moderate hypothermic circulatory arrest between 2001 and 2021. ATAAD was defined as the dissection of the ascending aorta with extension to the arch or to the descending aorta, regardless of the site of the primary intimal tear. Diagnosis was generally established with emergent computed tomographic (CT) angiography of the chest, abdomen, and pelvis. Bedside transthoracic echocardiography assessed pericardial effusion and left ventricular function. Patients also underwent transesophageal echocardiography in the operating room to evaluate heart valves and the potential need for concomitant procedures. The presence of PSCM was identified using CT scan or equivalent and was evaluated for the presence of signs of stroke or cerebral ischemia. Primary endpoint outcomes were the following: (1) in-hospital and 30-day mortality, (2) postoperative new neurological deficit, (3) mechanical ventilation hours, and (4) intensive care unit (ICU) length of stay.

### 2.2. Surgical Technique and Postoperative Management

The surgical technique and the protocol followed in our university hospital has been previously described [12]. Experienced senior surgeons performed all cases under general anesthesia with standard hemodynamic monitoring. After cross-clamping of the aorta, myocardial protection involved retrograde cold blood cardioplegia. To provide bilateral antegrade cerebral perfusion, oxygenated cold blood (18 °C) was delivered through a balloon catheter inserted into the arch vessels, ensuring controlled flow pressure at 50–60 mmHg. The extent of the intimal tear determined the surgical approach, including supracoronary ascending aortic replacement, total vs. hemi- arch replacement, frozen elephant trunk, and reimplantation of supra-aortic arteries. The need for associated coronary artery bypass grafting or a Conduit/Bentall procedure with reimplantation of coronary arteries versus a David operation was also determined according to assessment of disease extent. After anastomosis, intracardiac air was ruled out using transesophageal echocardiography. Once primary hemostasis was achieved, the chest was closed, and the patient was subsequently transferred to the cardiac intensive care unit (ICU) to receive standard postoperative care.

Neurological deficits were routinely assessed in patients every hour during their stay in the ICU and every eight hours after being transferred to the floor. If a new deficit was detected, a CT scan of the head was performed, followed by formal neurological evaluation and magnetic resonance imaging to confirm the diagnosis. Mechanical ventilation was gradually discontinued following a standard postoperative protocol, with the aim of achieving liberation as soon as possible. Tracheostomy was considered if weaning from mechanical ventilation and extubation were not achievable within 10–12 days after the surgery.

### 2.3. Statistical Analysis

Descriptive statistics were used to present the baseline characteristics, operative details, and postoperative outcomes of patients in both groups. The normality of continuous variables was assessed using the Kolmogorov–Smirnov and the Shapiro–Wilk tests. For normally distributed data, group differences were assessed using the T-test, while for non-normally distributed data, the Mann–Whitney U-test was employed. The results were reported as the median and interquartile range (IQR). Categorical data were summarized as absolute (n) and relative (%) frequencies and compared by Chi^2^-test or Fisher’s exact test. Survival was estimated using the Kaplan–Meier curves for right censored data and analyzed for differences between the PSCM and the non-CM groups by the log rank test. Cox regression analysis was employed to examine the risk factors associated with long-term survival. Variable selection was based on clinical relevance and forward selection. All tests were conducted 2-sided and a *p*-value of ≤0.05 was considered statistically significant. IBM SPSS Statistics for Windows (Version 27.0) was used for statistical analysis.

## 3. Results

### 3.1. Preoperative and Baseline Patients’ Characteristics

Of the total cohort, 82 patients (17.1%) had PSCM. The age, sex, and body mass index showed no significant differences between the groups. The patients with PSCM exhibited a higher logistic EuroSCORE I (median: 47, IQR [31, 64] vs. 24, IQR [15, 39]; *p* < 0.001) and had a higher prevalence of arterial hypertension (74.4% vs. 63.1%; *p* = 0.05). Although the EuroSCORE II was numerically higher in the PSCM group, the difference did not reach statistical significance (9.42 vs. 6.62; *p* = 0.44).

Interestingly, the patients in the PSCM group had a smaller aneurysmal diameter (48 mm, IQR [45, 53] vs. 52 mm, IQR [49, 60]; *p* = 0.048) compared to those without PSCM. Furthermore, the patients in the PSCM group were more likely to have undergone a previous cardiac surgery (7.3% vs. 2%; *p* = 0.02). Table 1 provides a summary of the baseline characteristics of the included patients. In terms of the laboratory data, we observed that the C-reactive protein levels were significantly higher in the PSCM group prior to surgery (6.5 mg/dL, IQR [2.1, 52.6] vs. 4.25 mg/dL, IQR [1.2, 21.5]; *p* = 0.01).

### 3.2. Intraoperative Details

The operative, bypass, cardioplegia, and aortic cross clamp times were similar between both groups, as shown in Table 2. The patients without PSCM underwent more Bentall surgeries (8.5% vs. 22.4%; *p* = 0.004) and had more aortic valve replacements (6.1% vs. 21.6%; *p* = 0.001). The patients presenting with PSCM received more intraoperative packed red blood cells (4 units, IQR [2, 6] vs. 2 units, IQR [0, 5]; *p* < 0.001), fresh frozen plasma (3 units, IQR [0, 6] vs. 0 units, IQR [0, 4]; *p* = 0.003), and platelets (2 units, IQR [1, 2] vs. 2 units, IQR [1, 2]; *p* = 0.035). There were no significant differences observed in the choice of arterial or venous cannulation sites between both groups.

### 3.3. Postoperative Outcomes

Following the surgery, the patients who presented with PSCM had markedly longer ventilation hours (108.5 h, IQR [44, 277] vs. 43 h, IQR [16, 158], *p* < 0.001) and a significantly longer ICU length of stay (7 days, IQR [4, 13] vs. 5 days, IQR [2, 11]; *p* = 0.013). Additionally, the patients with preoperative PSCM had significantly higher rates of postoperative new neurological deficits (35.4% vs. 19.4%; *p* = 0.002). Moreover, they underwent more tracheotomies (35.4% vs. 20.7%; *p* = 0.004) and experienced more postoperative pneumonia (22% vs. 12.5%; *p* = 0.026). No significant difference was noted in terms of the postoperative inotrope requirement, chest tube drainage, or postoperative delirium between both groups. Table 3.

In terms of the laboratory data, we observed that patients in the PSCM group had higher levels of plasma potassium (5 mmol/L, IQR [4.7, 5.31]) compared to the patients without PSCM (4.8 mmol/L, IQR [4.5, 5.1]; *p* = 0.002) on the first postoperative day. Additionally, the PSCM group had higher levels of C-reactive protein (141.6 mg/dL, IQR [92, 194] vs. 102 mg/dL, IQR [42.5, 161.3]; *p* = 0.005) and a lower platelet count (111 × 10^9^/L IQR [90, 141] vs. 130 [104, 161]; *p* = 0.004) on the first postoperative day.

The PSCM group tended to have higher in-hospital mortality rates; however, the results were not statistically significant (19.8% vs. 15.3%; *p* = 0.32). Furthermore, no significant difference was observed in the 30-day mortality between both groups (22% vs. 17.2%; *p* = 0.31). Table 4 illustrates the differences in the cause-specific mortality between both groups.

**Figure 1:**
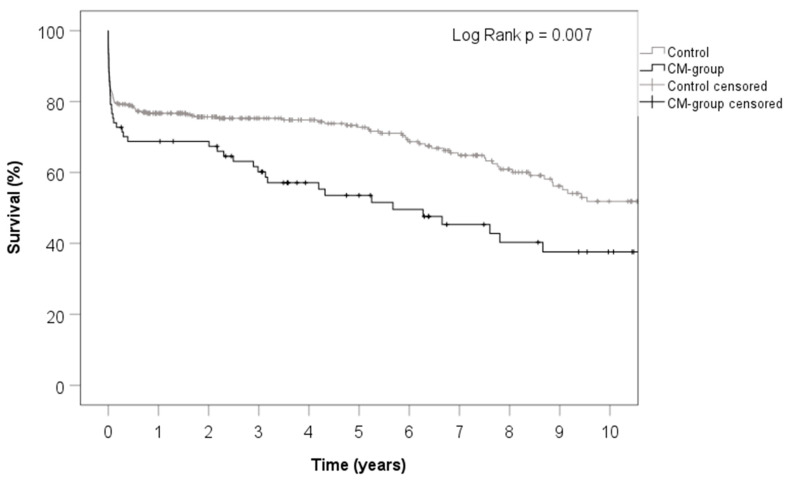
Kaplan–Meier curve illustrating long-term survival throughout follow-up. There is a significantly lower survival rate in patients who had PSCM after ten years of follow-up (38% vs. 52%, log-rank test *p* value = 0.007).

In the long term, there was a significant decline in survival in the PSCM group, as indicated by the KM curve (*p* = 0.007). Figure 1 shows the Kaplan–Meier analysis.

### 3.4. Risk Factors for Long-Term Mortality

In our multivariate Cox regression analysis, several significant risk factors associated with long-term mortality were identified. PSCM emerged as a significant risk factor (hazard ratio (HR) 1.75, 95%CI [1.20–2.53], *p* = 0.003). Additionally, factors such as ventilation upon admission and cardiopulmonary resuscitation within 48 h of admission were also found to be associated with a lower survival. Surprisingly, hypertension was shown as a protective factor against long-term mortality. (HR: 0.59, 95%CI [0.43–0.82], *p* = 0.001). Table 5 presents a summary of the variables that were tested in the Cox regression analysis, along with their respective hazard ratios.

## 4. Discussion

ATAAD is a complex and life-threatening condition characterized by high mortality rates and perioperative complications. The presence of malperfusion syndromes further aggravates these challenges and contributes to the poorer outcomes. Our study aimed to investigate the characteristics and outcomes of ATAAD patients who presented with CM.

In our large center experience, we observed that 17.1% of the patients diagnosed with ATAAD also presented with CM. This finding is consistent with previous estimates, which have reported CM occurring in approximately one sixth of ATAAD cases [11].

Furthermore, our results showed a trend indicating an association between PSCM and increased in-hospital mortality rates. Wang and colleagues [11] reported an average in-hospital mortality rate of around 20% in ATAAD patients presenting with CM. Our findings, showing an in-hospital mortality of 19.8% and a 30-day mortality of 22%, align with these previous studies. Although we did not observe a statistically significant difference compared to the patients without PSCM, the observed trends suggest a potential link between PSCM and a poorer prognosis. Interestingly, we found that the impact of PSCM on the patient survival becomes more evident in the long term, with significantly lower survival rates in the PSCM group.

Importantly, our findings highlight the significant impact of PSCM on the postoperative outcomes. For example, the patients with PSCM had significantly longer ventilation hours and intensive care unit stays compared to those without PSCM. This highlights the complex impact leading to respiratory insufficiency. Previous reports have also highlighted the intricate nature of postoperative respiratory complications in patients with CM, showing consistently prolonged durations of mechanical ventilation in patients with CM [13,14,15]. Furthermore, the prolonged ventilation can be mirrored by the higher rate of tracheotomies and the increased incidence of postoperative pneumonia in the CM group, indicating the severity of respiratory compromise in patients with CM and the challenges in weaning them off mechanical ventilation.

Several reports have emphasized the risk imposed by preoperative CM and the influence on the incidence of postoperative new neurological deficits [11,15,16,17,18,19]. Our results showed that 35.4% of the patients with PSCM experienced new postoperative neurological deficits. Wang and colleagues also showed that postoperative new neurological deficits can occur in up to 45.5% of patients who have PSCM [11]. Nonetheless, it is important to note that the preoperative CM can be difficult to categorize at admission and the accurate assessment of neurological symptoms is difficult, especially when patients are admitted intubated. Gomibuchi et al. [17] demonstrated that the presence of supra-aortic branch occlusion or stenosis is a significant risk factor for permanent neurological deficit (odds ratio: 7.66; *p* < 0.001), regardless of the preoperative neurological symptoms. On the other hand, Vendramin et al. [15] noted in their series that patients presenting with coma had the highest in-hospital mortality, regardless of the brain protection.

Despite the earlier debate on whether patients with preoperative CM should undergo prompt surgery [18], the current guidelines encourage no delay in surgery for those patients [6,7]. The systematic review by Wang showed that 54.3% of patients with preoperative CM experienced complete recovery or improvement in their neurological symptoms, while the symptoms remained the same in 27.1% of patients and worsened in 8.5% of patients. Similarly, Di Eusanio et al. [18] demonstrated an improved late survival and frequent reversal of neurologic deficits.

The extent of supra-aortic vessel involvement was reported as a significant moderator of poor neurological outcomes. Fukuhara and colleagues [16] showed that the extension of the dissection to the internal carotid artery was the worst predictor of poor outcomes when compared to common carotid involvement. In their experience, all patients with internal carotid artery occlusion developed cerebral edema and herniation syndromes, and died regardless of the management.

Several management strategies have been proposed to manage ATAAD patients presenting with PSCM and reduce the risk of neurological complications. Those include early aggressive direct carotid reperfusion before surgical repair [19], percutaneous stenting or endovascular repair [20], and extra-anatomic revascularization [17]. Moreover, a hemi-arch replacement was proposed as a strategy to manage patients with supra-aortic vessel dissection but no PSCM, still, such a strategy carried a higher risk of reoperation [21]. The optimal management approach for CM in ATAAD remains uncertain. Fukuhara et al. [16] emphasized the importance of performing neck computed tomography prior to surgery, as it can provide valuable information about the extent of dissection involving the supra-aortic vessels and guide the intraoperative management decisions for these cases.

Recent evidence has provided insights into the potential value of serum biomarkers in ATAAD patients. Besides elevated C-reactive protein, the lymphocyte-to-neutrophil ratio and lymphocyte-to-platelet ratio (LPR) have emerged as predictive markers of mortality [22,23]. Interestingly, a study conducted by Yuan et al. [4] identified a plasma potassium of >4.4 mmol/L as an independent predictor of mortality in ATAAD patients. Consistent with these findings, our study revealed that patients with PSCM exhibited elevated postoperative serum potassium levels, increased levels of C-reactive protein, and lower platelet counts. The exact role and clinical significance of these biomarkers in ATAAD warrant further investigation for a better understanding of their diagnostic and prognostic implications.

In our multivariate Cox regression analysis, we identified PSCM as a significant risk factor for long-term mortality, emphasizing its significant impact posed on patients’ long-term survival. Additionally, factors such as ventilation upon admission and cardiopulmonary resuscitation within 48 h of admission were associated with lower survival rates, which could be rationally attributed to the critical baseline morbidity in those patients. Intriguingly, our study revealed that hypertension has a protective effect against long-term mortality. Previous studies have suggested that a known history of hypertension may contribute to the earlier diagnosis and management of patients with ATAAD, potentially leading to lower mortality rates [24,25]. This earlier recognition and management may also mitigate the impact of CM on brain tissue viability in patients with PSCM. Moreover, hypertension could theoretically enhance the collateral circulation and reduce the damage to the penumbral tissue [26], potentially reducing the overall impact of CM on patients’ long-term survival. Nevertheless, this theory warrants further investigation to better understand its potential role in this specific subgroup of ATAAD patients.

Further research is also warranted to explore the optimal approaches for addressing CM in patients with ATAAD and minimizing its impact on patient outcomes.

## 5. Strengths, Limitations, and Future Recommendations

Our study provides valuable real-world data that reflect the clinical practice and patient populations encountered in daily practice. It is important to acknowledge several limitations that should be considered when interpreting the results. Firstly, being a retrospective observational study, there is the potential for an inherent confounding bias that may impact the generalizability of our findings. Secondly, the complex nature of the patient pathology in ATAAD makes it challenging to draw definitive conclusions regarding the specific impact of CM on outcomes, particularly considering the potential influence of other malperfusion syndromes. Moreover, CT scans were employed to detect the presence of PSCM; therefore, our data do not provide a clear cause of CM whether due to a dissection-related artery obstruction or severe hemodynamic changes that resulted in a stroke. Future research may benefit from performing a preoperative perfusion CT or an intraoperative transcranial Doppler to obtain more conclusive evidence on the presence of PSCM.

It is important to mention that the postoperative parameters discussed may not be solely attributed to CM but could reflect the overall complexity of cases. It is crucial to exercise caution and to consider these limitations when interpreting the results. Further research, including prospective studies with larger sample sizes and more comprehensive assessments, is warranted to better elucidate the association between CM and outcomes in ATAAD patients.

## 6. Conclusions

PSCM in patients with ATAAD were associated with longer ventilation hours, the ICU length of stay, and a higher incidence of postoperative new neurological deficits. Moreover, the long-term survival outcomes were significantly poorer in patients presenting with PSCM. These results highlight the need for early recognition and appropriate management of neurologic deficits in ATAAD to improve the postoperative outcomes and long-term survival.

## Figures and Tables

**Table 1:** Summary of the baseline characteristics and preoperative data of the included patients.

Variable	Total (n = 480)	No PSCM (n = 398/82.9%)	PSCM (n = 82/17.1%)	*p*-Value
Age (years)	63 (53; 73)	62 (53; 73)	66 (56; 73)	0.077
Female gender	170 (35.4%)	139 (34.9%)	31 (37.8%)	0.619
Body mass index [kg/m^2^]	26.3 (24; 29.3)	26.3 (24; 29.3)	26.3 (23.4; 28.6)	0.530
Logistic EuroScore I	27 (16; 42)	24 (15; 39)	47 (31; 64)	**<0.001**
EuroScore II	6.86 (4.07; 14.14)	6.62 (3.92; 13.27)	9.42 (4.96; 14.66)	0.44
LVEF [%]	60 (55; 70)	60 (55; 70)	60 (55; 70)	0.804
Aneurysm Diameter [mm]	52 (47; 60)	52 (49; 60)	48 (45; 53)	**0.048**
DeBakey I	380 (79.3%)	314 (79.1%)	66 (80.5%)	0.776
DeBakey II	100 (20.7%)	84 (20.9%)	16 (19.5%)	0.776
Arterial hypertension	312 (65%)	251 (63.1%)	61 (74.4%)	0.050
IDDM	6 (1.3%)	4 (1%)	2 (2.4%)	0.275
Acute kidney failure	9 (1.9%)	8 (2%)	1 (1.2%)	1.000
Chronic kidney failure	51 (10.6%)	43 (10.8%)	8 (9.8%)	0.774
COPD	33 (6.9%)	30 (7.5%)	3 (3.7%)	0.206
PAD	15 (3.1%)	12 (3%)	3 (3.7%)	0.729
CAD	75 (15.6%)	62 (15.6%)	13 (15.8%)	0.537
Bicuspid aortic valve	23 (4.8%)	22 (5.6%)	1 (1.2%)	0.297
Marfan syndrome	13 (2.7%)	10 (2.5%)	3 (3.7%)	0.473
Previous PCI	33 (6.9%)	30 (7.6%)	3 (3.7%)	0.213
Previous thoracic intervention	41 (8.5%)	30 (7.5%)	11 (13.4%)	0.083
Previous cardiac surgery	14 (2.9%)	8 (2%)	6 (7.3%)	**0.020**
Pericardial tamponade	78 (16.3%)	65 (16.4%)	13 (15.9%)	0.901
Acute MI (≤48 h)	15 (3.1%)	13 (3.3%)	2 (2.4%)	1.000
Cardiogenic shock	33 (6.9%)	29 (7.3%)	4 (4.9%)	0.430
CPR (≤48 h)	40 (8.3%)	37 (9.3%)	3 (3.7%)	0.093
ICU transfer	71 (14.8%)	62 (15.6%)	9 (11%)	0.282
Ventilated on admission	52 (10.9%)	44 (11.1%)	8 (9.8%)	0.725
Atrial fibrillation	56 (11.7%)	43 (10.8%)	13 (15.9%)	0.195
Aortic valve regurgitation	157 (33.7%)	131 (33.8%)	26 (33.3%)	0.302
C-reactive protein (mg/dL)	4.75 (1.28; 23.7)	4.25 (1.2; 21.5)	6.5 (2.1; 52.6)	**0.014**

PSCM: preoperative signs of cerebral malperfusion, EuroScore: European System for Cardiac Operative Risk Evaluation; COPD: chronic obstructive pulmonary disease; LVEF: left ventricular ejection fraction; IDDM: insulin dependent diabetes mellitus; PAD: peripheral arterial disease; CAD: coronary artery disease; PCI: percutaneous coronary intervention; MI: myocardial infarction; CPR: cardiopulmonary resuscitation; ICU: intensive care unit; Bold: Statistically significant.

**Table 2:** Intraoperative details.

Variable	Total (n = 480)	No PSCM (n = 398/82.9%)	PSCM (n = 82/17.1%)	*p*-Value
Surgery duration [min]	281 (228; 347)	280 (227; 349)	289 (230; 335)	0.993
CPB [min]	168 (135; 215)	171 (135; 224)	160 (139; 193)	0.132
Cross clamp duration [min]	95 (72; 137)	96 (72; 140)	90 (71; 118)	0.166
Circulatory arrest [min]	35 (26; 51)	34 (26; 51)	39 (28; 55)	0.199
RBC [unit]	2 (0; 6)	2 (0; 5)	4 (2; 6)	**<0.001**
FFP [unit]	0 (0; 6)	0 (0; 4)	3 (0; 6)	**0.003**
Platelets [unit]	2 (1; 2)	2 (1; 2)	2 (1; 2)	**0.035**
Supracoronary aortic replacement ONLY	202 (42.1%)	163 (41%)	39 (47.6%)	0.270
Hemi-arch	119 (24.8%)	96 (24.1%)	23 (28%)	0.453
Total-arch	72 (15%)	61 (15.3%)	11 (13.4%)	0.659
Conduit/Bentall	96 (20%)	89 (22.4%)	7 (8.5%)	**0.004**
David	29 (6%)	26 (6.5%)	3 (3.7%)	0.447
Elephant-trunk	13 (2.7%)	11 (2.8%)	2 (2.4%)	1.000
Aortic valve replacement	**91 (19%)**	**86 (21.6%)**	**5 (6.1%)**	**0.001**
CABG	37 (7.7%)	34 (8.5%)	3 (3.7%)	0.131
Arterial cannulation site				
Femoral artery	81 (17.3%)	62 (16%)	19 (23.5%)	0.283
Ascending aorta	93 (19.9%)	74 (19.1%)	19 (23.5%)	0.283
Aortic arch	13 (2.8%)	10 (2.6%)	3 (3.7%)	0.283
Subclavian artery	2 (0.4%)	2 (0.5%)	0 (0%)	0.283
Apex	5 (1.1%)	4 (1%)	1 (1.2%)	0.283
Pulmonary vein	274 (58.5%)	235 (60.7%)	39 (48.1%)	0.283
Venous cannulation site				
Right Atrium	455 (97.2%)	378 (97.7%)	77 (95.1%)	0.086
bicaval	4 (0.9%)	4 (1%)	0 (0%)	0.086
Femoral vein	9 (1.9%)	5 (1.3%)	4 (4.9%)	0.086

PSCM: preoperative signs of cerebral malperfusion; CPB: cardiopulmonary bypass; RBC: red blood cells; FFP: fresh frozen plasma; CABG: coronary artery bypass grafting. Bold: Statistically significant.

**Table 3:** Postoperative outcomes.

Variable	Total (n = 480)	No PSCM (n = 398/82.9%)	PSCM (n = 82/17.1%)	*p*-Value
Postoperative inotropic therapy	64 (14%)	54 (14.3%)	10 (12.7%)	0.213
48 h chest tube output [mL]	910 (500; 1650)	950 (500; 1700)	900 (500; 1600)	0.987
24 h RBC [unit]	0 (0; 2)	0 (0; 2)	1 (0; 2)	0.525
24 h FFP [unit]	0 (0; 4)	0 (0; 4)	0 (0; 4)	0.894
24 h Platelets [unit]	0 (0; 0)	0 (0; 0)	0 (0; 1)	0.424
Total RBC given [unit]	3 (0; 8)	2 (0; 8)	4 (0; 8)	0.314
Total FFP [unit]	1.5 (0; 6)	2 (0; 6)	0 (0; 4)	0.189
Total platelets [unit]	0 (0; 2)	0 (0; 2)	0 (0; 1)	**0.042**
Ventilation [h]	17 (60; 189)	43 (16; 158)	108.5 (44; 277)	**<0.001**
ICU stay [d]	5 (2; 11)	5 (2; 11)	7 (4; 13)	**0.013**
ICU re-admission	39 (8.2%)	33 (8.4%)	6 (7.3%)	0.737
Reintubation	77 (16.3%)	63 (16.1%)	14 (17.1%)	0.830
Tracheotomy	110 (23.2%)	81 (20.7%)	29 (35.4%)	**0.004**
Delirium	93 (19.8%)	81 (20.9%)	12 (14.6%)	0.197
MI	6 (1.3%)	6 (1.5%)	0 (0%)	0.596
New neurological deficits	105 (22.2%)	76 (19.4%)	29 (35.4%)	**0.002**
CPR	29 (6.1%)	25 (6.4%)	4 (4.9%)	0.603
Pneumonia	67 (14.2%)	49 (12.5%)	18 (22%)	**0.026**
Sepsis	21 (4.4%)	16 (4.1%)	5 (6.1%)	0.385
TEVAR(EVAR)	31 (6.5%)	27 (6.9%)	4 (4.9%)	0.522
Re-thoracotomy	89 (18.7%)	78 (19.7%)	11 (13.4%)	0.180
Wound healing deficits	7 (1.5%)	6 (1.5%)	1 (1.2%)	1.000
AKI KDIGO	102 (21.7%)	85 (21.9%)	17 (20.7%)	0.815
Postoperative AF	46 (9.8%)	37 (9.5%)	9 (11.1%)	0.654
New pacer	23 (4.9%)	20 (5.1%)	3 (3.7%)	0.780
Postoperative C-reactive protein (mg/dL)	110.5 (47; 173.1)	102 (42.55; 161.38)	141.6 (91.8; 193.65)	**0.005**
Postoperative platelets count	127.5 (101.25; 157)	130 (104; 161)	111 (90; 141)	**0.004**

PSCM: preoperative signs of cerebral malperfusion; RBC: red blood cells; FFP: fresh frozen plasma; ICU: intensive care unit; MI: myocardial infarction; CPR: cardiopulmonary resuscitation; TEVAR: thoracic endovascular aortic repair; AKI: acute kidney injury; AF: atrial fibrillation. Bold: Statistically significant.

**Table 4:** Early overall and cause-specific mortality.

Variable	Total (n = 480)	No Cerebral Malperfusion (n = 398/82.9%)	Cerebral Malperfusion (n = 82/17.1%)	*p*-Value
In-hospital mortality	77 (16.1%)	61 (15.3%)	16 (19.8%)	0.323
Cause of death				
Cardiac	44 (50%)	34 (49.3%)	10 (52.6%)	0.064
Cerebrovascular	8 (9.1%)	4 (5.8%)	4 (21.1%)	0.064
Sepsis	4 (4.5%)	2 (2.9%)	2 (10.5%)	0.064
Multi-organ failure	28 (31.8%)	25 (36.2%)	3 (15.8%)	0.064
Unknown	4 (4.5%)	4 (5.8%)	0 (0%)	0.064
Surgery till death [d]	3 (1; 12)	3 (1; 11)	6 (1; 16)	0.223
7-day mortality	56 (11.8%)	46 (11.7%)	10 (12.2%)	0.900
30-day mortality	86 (18%)	68 (17.2%)	18 (22%)	0.310

**Table 5:** Cox regression analysis on risk factors for long-term survival.

Risk Factor	Hazard Ratio	95% Confidence Interval	*p*-Value
Age (years)	1.041	[1.026–1.057]	**<0.001**
Female gender	0.949	[0.682–1.321]	0.758
PSCM	1.747	[1.204–2.534]	**0.003**
Arterial hypertension	0.595	[0.433–0.817]	**0.001**
PAD	1.890	[0.917–3.896]	0.085
Ventilated on admission	1.677	[1.050–2.679]	**0.031**
CPR (<48 h)	2.610	[1.622–4.202]	**<0.001**
CPB (min)	1.007	[1.004–1.009]	**<0.001**
RBC (unit)	1.043	[1.000–1.088]	**0.048**

PSCM: preoperative signs of cerebral malperfusion, PAD: peripheral artery disease, CPR: cardiopulmonary resuscitation, CPB: cardiopulmonary bypass time, RBC: red blood cell transfusion. Bold: Statistically significant.

## Data Availability

The data presented in this study are available on request from the corresponding author. The data are not publicly available due to institutional policies.
